# Identification and Expression Analysis of the *NPF* Genes in Cotton

**DOI:** 10.3390/ijms232214262

**Published:** 2022-11-17

**Authors:** Qiang Dong, Guoxin Wang, Asif Iqbal, Noor Muhammad, Xiangru Wang, Huiping Gui, Hengheng Zhang, Mirezhatijiang Kayoumu, Xiaotong Li, Xiling Zhang, Meizhen Song

**Affiliations:** 1 State Key Laboratory of Cotton Biology, Institute of Cotton Research of Chinese Academy of Agricultural Sciences, Anyang 455000, China; 2 Western Agricultural Research Center of Chinese Academy of Agricultural Sciences, Changji 831100, China; 3 Zhengzhou Research Base, State Key Laboratory of Cotton Biology, School of Agricultural Sciences, Zhengzhou University, Zhengzhou 450001, China

**Keywords:** cotton, *NPF* genes, expression pattern, N deficiency conditions

## Abstract

The NPF (NITRATE TRANSPORTER 1/PEPTIDE TRANSPORTER FAMILY) transports various substrates, including nitrogen (N), which is essential for plant growth and development. Although many NPF homologs have been identified in various plants, limited studies on these proteins have been reported in cotton. This study identified 75, 71, and 150 NPF genes in *Gossypium arboreum*, *G. raimondii,* and *G. hirsutum*, respectively, via genome-wide analyses. The phylogenetic tree indicated that cotton *NPF* genes are subdivided into eight subgroups, closely clustered with *Arabidopsis* orthologues. The chromosomal location, gene structure, motif compositions, and cis-elements have been displayed. Moreover, the collinearity analysis showed that whole-genome duplication event has played an important role in the expansion and diversification of the *NPF* gene family in cotton. According to the transcriptome and qRT-PCR analyses, several *GhNPFs* were induced by the nitrogen deficiency treatment. Additional functional experiments revealed that virus-induced silencing (VIGS) of the *GhNPF6.14* gene affects the growth and N absorption and accumulation in cotton. Thus, this study lays the foundation for further functional characterization of *NPF* genes in cotton.

## 1. Introduction

Nitrogen (N) is essential for the survival of all living organisms; it is an important component of amino acids, nucleic acids, and other biologically significant compounds [1]. Nitrate (NO_3_^−^), ammonium (NH_4_^+^), and organic nitrogen compounds are the principal forms of soil N. Among these, NO_3_^−^ is the predominant form and essential for plant growth and development [2]. Nitrate transporters primarily mediate NO_3_^−^ absorption from the soil and its translocation to other parts of the plant. Four protein families, including nitrate transporter 1/peptide transporter family (NPF), nitrate transporter 2 (NRT2), chloride channel family (CLC), and progressively activating anion channel (SLAC/SLAH), are involved in NO_3_^−^ transportation [3,4]. Several NPFs have been proposed to function as dual-affinity transporters active in high-affinity and low-affinity transport systems [3,5]. The NPF family is one of the largest transporter families in plants [6], and phylogenetic analysis shows that *NPF* genes can be separated into eight subfamilies [7].

The first plant *NPF* gene to be functionally characterized was *NPF6.3/NRT1.1/CHL1* [8]. In *Arabidopsis*, *AtNPF6.3* plays an important role in NO_3_^−^ absorption and root-to-shoot nitrate translocation through bidirectional transport activities [9]. Several other *Arabidopsis* and rice *NPF* genes are reportedly involved in plant growth and development through signal transduction and root development [4]. It has also been reported that in addition to nitrate transport activities, some nitrate transporters are also involved in plant stress responses. For example, *AtNPF7.3* [10] and *AtNPF7.2* transport nitrate through xylem vessels [11] and facilitate the xylem nitrate unloading process under stress conditions [12]. Recent studies demonstrated that OsNPF7.9 expresses preferentially in xylem parenchyma cells of vasculature tissues and is essential for balancing rice growth and stress tolerance [13]. Thus, *NPFs* are crucial for plant growth and development; however, little is known about their gene functions in plants other than rice and *Arabidopsis*.

Cotton (*Gossypium* spp.) is an important economic crop, accounting for 35% of natural fiber globally [14]. Cotton requires higher amounts of N for appropriate growth and development, and N deprivation in the soil may inhibit its growth, yield, and fiber quality [15,16,17]. Therefore, it is crucial to understand the molecular mechanisms of N uptake and utilization in cotton. Although the functions of *NPF* family genes have been characterized in several plants, including rice [18], poplar [19], wheat [20,21,22], legumes [23], apple [24], sugarcane [25], oilseeds [26], spinach [27], tea plant [28], and agave [29], little is known about cotton *NPF* family genes. To elucidate the activities of cotton *NPF* genes, we analyzed the entire *NPF* gene family of the three *Gossypium* species, including two diploid cotton species (*G. raimondii* and *G. arboreum*) and one tetraploid species (*G. hirsutum*). Here, we extensively examined the phylogenetic distribution, chromosomal location, gene structure, preserved motifs, duplication pattern, and selective stress of the *NPF* genes. Furthermore, RNA sequencing data were used to investigate the expression profiles of the cotton *NPF* genes, and quantitative real-time PCR was used to evaluate how the genes responded to low N supply. Thus, this study serves as the basis for the functional characterization of *GhNPFs*.

## 2. Results

### 2.1. Identification and Phylogenetic Analysis of NPF Genes in the Three Gossypium Species

A total of 75, 71, and 150 putative NPF protein sequences were identified from the *G. raimondii*, *G. arboreum*, and *G. hirsutum* genomes, respectively. The cotton NPF genes were named NPFX.Y according to the previously published guidelines, where X denotes the subfamily and Y denotes the individual member within the subfamily [7]. We also identified the locus ID, chromosome, start and stop codons, strand polarity, gene length, CDS length, protein length, estimated protein molecular weights, and isoelectric points for each member of the *NPF* family. Each cotton *NPF* gene typically encoded 261–674 amino acids (aa), with molecular weights ranging from 29.27 to 75.36 kDa, while the theoretical pI was between 4.99 and 9.56, as shown in Table S1.

**Figure 1:**
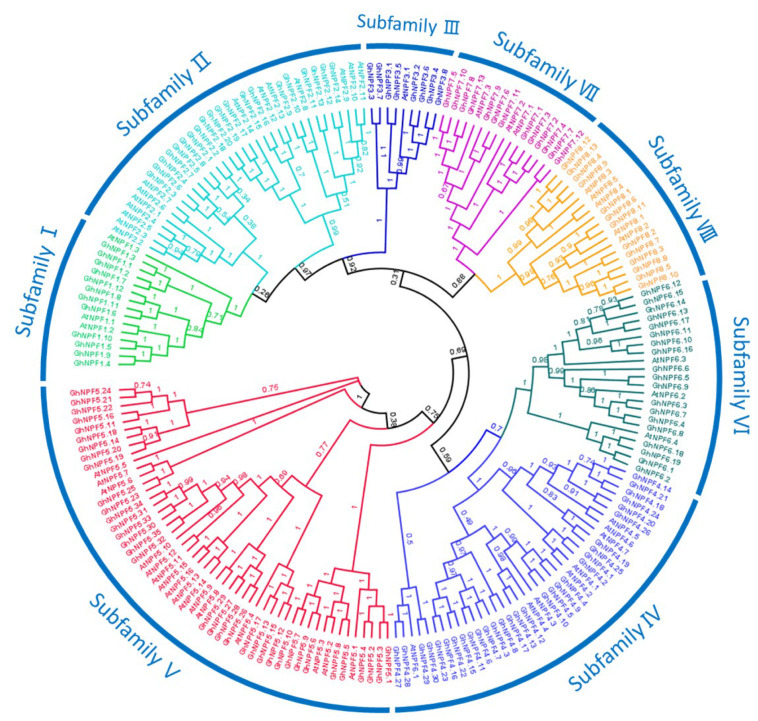
Phylogenetic tree and subgroup classification of NPF proteins in *A. thaliana* and *Gossypium hirsutum*. The numbers at the nodes of the phylogenetic tree indicate the bootstrap values expressing branching probability per 1000 replicates; the bootstrap values of the confidence levels are shown as percentages.

A phylogenetic tree was created, and the results showed that the identified cotton *NPF* genes could be further divided into 8 subgroups (I to VIII), as previously reported. In the tetraploid cotton, *G. hirsutum*, subfamilies IV and V were the largest, containing 30 and 35 *NPF* members, respectively, while subfamily III was the smallest, with only seven *NPF* members (Figure 1). However, in the diploid cotton species *G. arboreum* and *G. raimondii*, subfamily V was the largest, with the former containing 16 and the latter, 14 *NPF* members (Figure S1).

### 2.2. Gene Structures and Conserved Motifs Analysis

**Figure 2:**
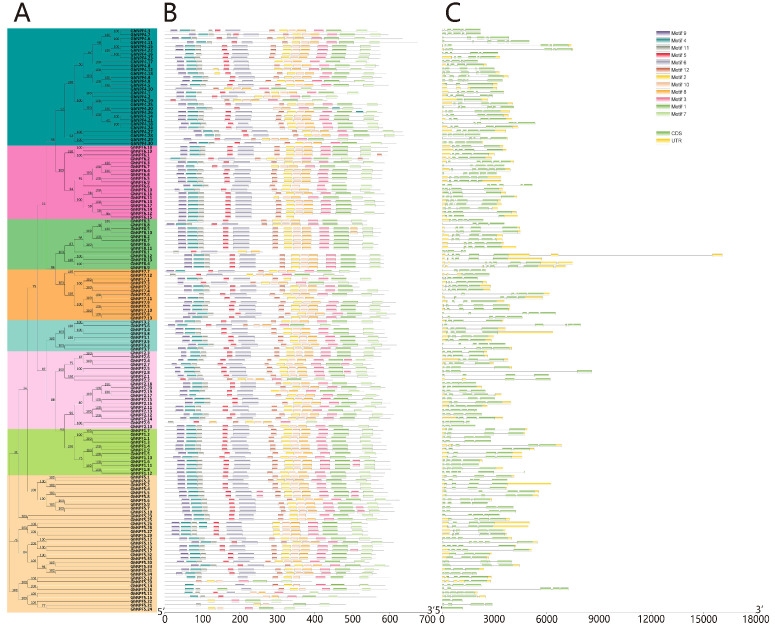
Conservative motifs and exon–intron organization of *NPF* gene family from Gossypium hirsutum. (**A**) Phylogenetic tree of *NPF* gene family obtained according to NJ method in MEGA software. (**B**) Conservative motifs of NPF proteins. The 12 conserved motifs in GhNPF proteins are indicated by multiple-colored boxes. (**C**) Exon–intron structures of *NPF* gene family.

The structures of 150 *NPF* genes obtained from *G. hirsutum* were analyzed using the GSDS web server to obtain their evolutionary links. The analysis revealed that the exon numbers were highly diverse among the *GhNPFs* and ranged from one to eight (Figure 2C). Most members of subfamilies II, III, VI, and VII contained four or five exons. A total of 58 (38.6%) *GhNPF* genes had 5 exons, accounting for the largest number of exons among the analyzed *GhNPF* genes, followed by 50 (33.3%), 25 (16.7%), 9 (6%), 5 (3.3%), 3 (2%), and 1 (0.6%) gene with 4, 6, 3, 2, 8, and 1 exon, respectively. In general, *NPF* genes in the same group exhibited similar exon–intron architectures.

The different motifs (including the conserved ones) of the *GhNPF* family and their subfamilies were identified using the MEME Suite motif analysis (Figure 2B and Figure S2). We found that all NPF proteins have 4 to 12 distinct motifs, and most members of the same subfamily display comparable motif construction. Among the 150 GhNPF proteins, nine truncated ones lacked the N-terminus (Motif9-Motif4-Motif11), and five other truncated members lacked the C-terminus (Motif3-Motif7-Motif1). Most GhNPF proteins contained motifs 4, 6, 7, and 8 within their conserved domains. Moreover, 92% of the GhNPF proteins had motif 1, while 94.7% had motif 2. Motif 3 existed in 94.7% of the GhNPF proteins. This finding shows that the sequences of the *GhNPF* gene family are highly conserved.

### 2.3. Chromosomal Position, Duplication, and Collinearity Analysis

The tetraploid cotton (*G. hirsutum*) chromosomes showed diversity in their *NPF* genes. We found that chromosome ChrA05 and its homolog ChrD05 had the highest gene-loci density containing *NPF* genes. However, ChrA11, ChrD1, and ChrD11 had the lowest gene-loci density, containing only two *NPF* genes. The diploid cotton species (*G. arboreum* and *G. raimondii*) showed variation in their chromosomal NPF genes, with *G. arboreum* having the highest gene loci (9) on chromosome A05 and *G. raimondii* having the loci (11) on chromosome D09 (Figure S3). These results indicated that *NPFs* are irregularly distributed across different chromosomal locations and have substantial conservation between the A and D genomes.

**Figure 3:**
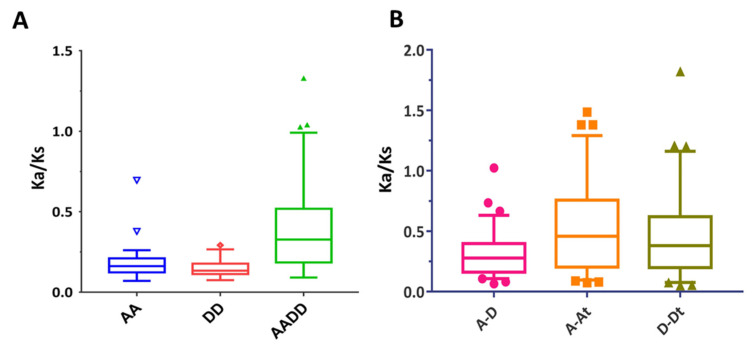
Ratios of non-synonymous to synonymous substitutions (Ka/Ks) in *NPF* genes. (**A**) Ka/Ks ratios of intraspecific duplicated gene pairs in *G. arboreum* (AA), *G. raimondii* (DD), and *G. hirsutum* (AADD); (**B**) Ka/Ks ratios of orthologous gene pairs between *G. arboreum* (AA) and *G. raimondii* (DD), *G. arboreum* (AA) and At sub-genome of *G. hirsutum*, *G. raimondii* (DD), and Dt sub-genome of *G. hirsutum*.

To understand the expansion pattern of the *NPF* gene family in cotton, we investigated the intraspecific duplication events of the *NPF* gene. We found 23, 12, and 69 duplicated gene pairs in *G. arboreum*, *G. raimondii*, and *G. hirsutum*, respectively (Table S2a), and only four pairs were linked to segmental duplication events in *G. arboreum*. However, all duplicated gene pairs were associated with tandem duplication events in *G. raimondii* and *G. hirsutum*. Furthermore, we evaluated the Ka/Ks ratio of each duplicated gene pair to analyze their molecular evolutionary rates. The results showed that most ratio values of the intraspecific duplicated gene pairs ranged from 0.09 to 1.33 (Figure 3A, Table S2a), and only four gene pairs had molecular evolutionary rates over 1. These four gene pairs included *GhNPF4.5*–*GhNPF4.10* (1.04), *GhNPF5.19*–*GhNPF5.20* (1.33), and *GhNPF5*.*21*–*GhNPF5*.*24* (1.03). These *NPF* duplicated gene pairs were subjected to purifying selection pressure.

**Figure 4:**
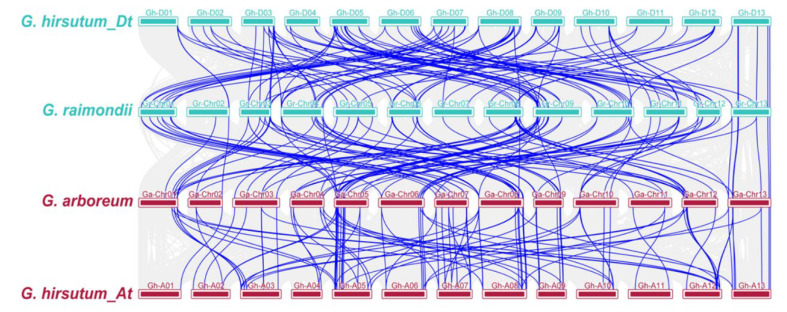
Synteny and collinearity relationships among *G. arboreum* (AA), *G. hirsutum* (At and Dt sub-genomes), and *G. raimondii* (DD). Blue and gray lines indicate *NPF* homologous gene pairs and collinear genes in the whole genome, respectively.

Furthermore, the collinearity and interspecific orthologous gene pairs were identified to investigate the evolution of the *NPF* genes in the three Gossypium species. We identified 65 collinear gene pairs in a comparison between the A and D genomes, 70 between the At sub-genome and the A genome and 63 between the Dt sub-genome and the D genome (Figure 4, Table S2b). The presence and relatedness of *NPF* genes among the three *Gossypium* species indicated that some *NPF* genes might have been lost during the speciation of *G. hirsutum*. Only 12 orthologous gene pairs exhibited positive selection, as shown by their Ka/Ks ratio, which was higher than 1 (Figure 3B, Table S2b). Most orthologous gene pairs had a Ka/Ks ratio of less than one, implying that purifying selection limited the functional divergence of *NPF* genes after duplications and polyploidization.

### 2.4. Cis-Acting Elements Analysis of GhNPFs

To elucidate the expression regulatory mechanisms of *GhNPF* genes, we determined the 1500-bp upstream sequences from the start codons of the *GhNPF* genes using the PLACE database. The results showed cis-elements related to hormonal signalling and stress responses (Figure S4 and Table S3). Several other cis-elements were also involved in stress responses, whereby 52 *GhNPFs* contained low-temperature responsiveness elements (LTRs) and 62 *GhNPFs* had drought-inducible elements (MBSs). Moreover, 118 *GhNPFs* contained the anaerobic induction elements (AREs), and elements regulating zein metabolism (O2-site) were contained in 70 *GhNPFs*, while meristem expression elements (CAT-box) were present in 45 *GhNPFs*. The defense-acting components and those regulating cis-adaptation to stress (TC-rich repeats) were contained in 55 *GhNPFs*, and all *GhNPFs* exhibited light responsiveness elements (Box4, G-Box, GT1-motif). We also identified five categories of hormone-related response components in *GhNPFs*. These included abscisic acid-responsive elements (ABRE) in 105 *GhNPFs*, auxin-responsive elements (TGA-element and AuxRR-core) in 47 *GhNPFs*, gibberellin-responsive elements (TATC-box, P-box, and GARE-motif) in 70 *GhNPFs*, and salicylic acid-responsive elements (TCA-element and SARE) in 70 *GhNPFs*. Therefore, these results support the theory that *GhNPF* genes are important in various biotic–abiotic and hormone signaling processes.

### 2.5. Expression Patterns Analysis of the GhNPF Genes

Various *NPF* gene families have been reported to participate in plant growth and development in different ways [21,30]. Transcriptome data of the TM−1 cultivar [31] were used to investigate the expression patterns of *GhNPF* genes in different tissues under different abiotic treatments. As shown in Figure S5 and Table S4, forty-seven *GhNPF* genes were found in all tissues, while three *GhNPF* genes (*GhNPF6.11*, *GhNPF7.1*, and *GhNPF8.1*) were only found in some tissues, indicating that these genes may be functionally redundant in *G. hirsutum*. Moreover, seven *GhNPFs* were highly expressed in the roots, implying their possible involvement in nitrate uptake. Another group of *GhNPFs* (*GhNPF4.8* and *GhNPF6.18*) were highly expressed in stems and leaves, indicating their association with nitrate phloem loading and intracellular transport. Additionally, eleven *GhNPFs* (including *GhNPF2.15* and *GhNPF3.4*) were specifically expressed in the pistil or stamen. Several other *GhNPFs* also showed significant expression levels during the growth of ovules (from −3 to 35 DPA) and fibers (5, 10, and 25 DPA), suggesting their significance in ovule and fiber development.

As shown in Figure S6 and Table S5, most *GhNPF* genes were up or downregulated in response to various treatments. We found that 43 *GhNPFs* were upregulated under the different stress treatments; however, *GhNPF4.9*, *GhNPF4.12*, and *GhNPF8.10* were downregulated in all treatments. Moreover, *GhNPF2.12, GhNPF2.14, GhNPF3.4, GhNPF3.8, GhNPF4.24, GhNPF6.4, GhNPF6.8, GhNPF6.18,* and *GhNPF6.19* were significantly upregulated under the four stress treatments. As shown in Figure S6, the up-regulation of some *GhNPFs* was stress-specific. These included *GhNPF6.2* under heat stress, *GhNPF8.12* and *GhNPF5.33* under drought stress, *GhNPF5.5* and *GhNPF4.27* under salt stress, and *GhNPF7.13* under cold stress. However, some *GhNPFs* were only upregulated at particular time points in some treatments; for example, *GhNPF7.9* was elevated at 6 h under salt stress, whereas *GhNPF4.20* and *GhNPF4.26* were increased at 6 h under drought stress.

### 2.6. GhNPF Genes Expression Pattern under N Deficiency Conditions

To reveal the expression patterns of *GhNPF* genes under N deficiency conditions, we moved the third-leaf stage seedlings into a 0.2 mM nitrate solution for treatment and a 2.0 mM nitrate solution as the control. Using expression patterns (Figures S5 and S6) and cotton N deficiency RNA-seq data (Table S6), we selected 54 *NPF* genes to determine their responses to low N stress.

**Figure 5:**
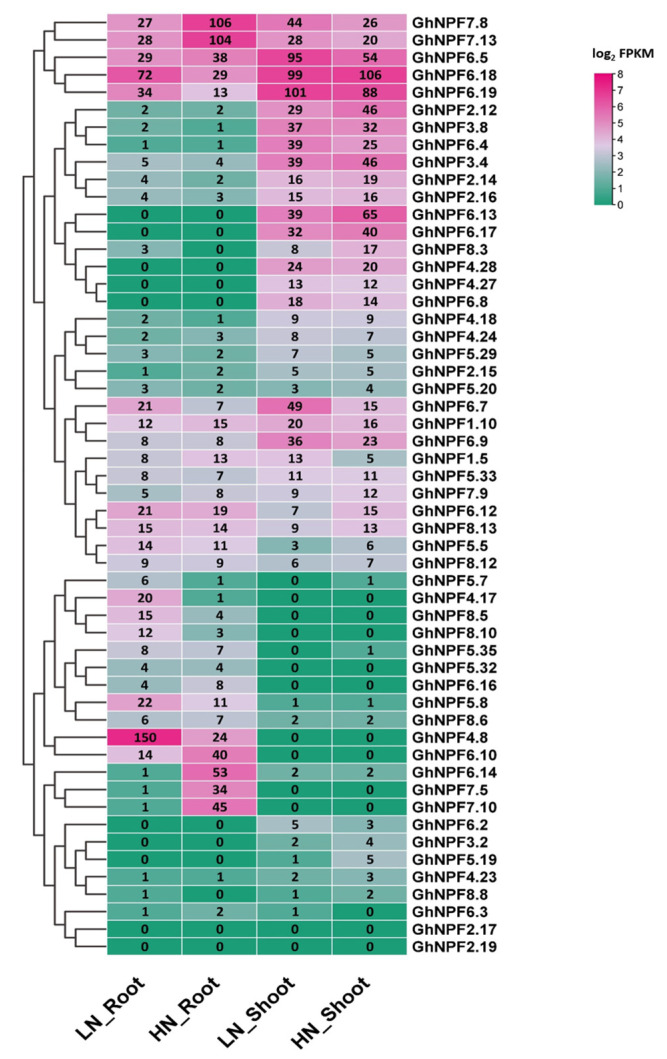
Expression profiles of 54 *GhNPF* family genes under N deficiency treatments. The expression data were obtained from RNA-seq data and shown as log_2_FPKM values.

As shown in Figure 5, the expression levels of *GhNPF4.8, GhNPF4.17, GhNPF6.18,* and *GhNPF6.19* were significantly upregulated, while *GhNPF6.14, GhNPF7.5*, and *GhNPF7.10* were significantly inhibited in the roots under N deficiency. In the shoots, four *GhNPF* genes (*GhNPF6.5, GhNPF6.7, GhNPF6.9*, and *GhNPF7.8*) were upregulated, while three *GhNPF* genes (*GhNPF2.12, GhNPF6.13*, and *GhNPF6.17*) were downregulated under N deficiency treatment.

**Figure 6:**
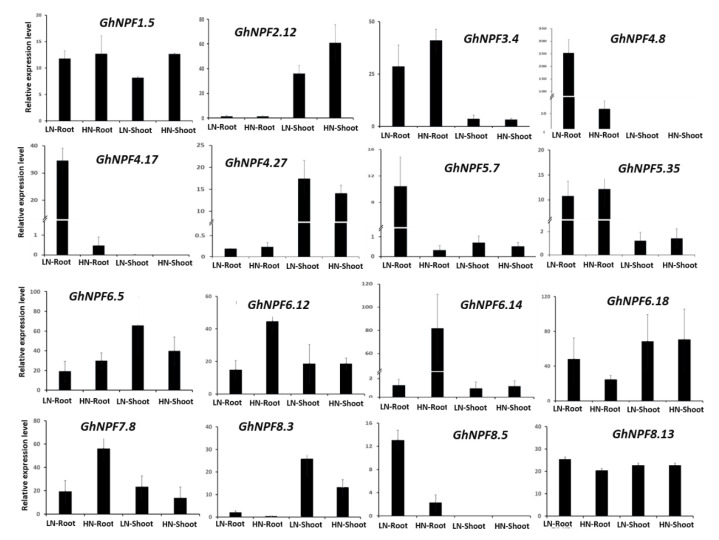
Relative expression patterns of 16 *GhNPF* genes under N deficiency treatments were analyzed by qRT-PCR. Error bars indicate the standard deviations of three independent experiments.

Furthermore, we selected 16 representative *GhNPF* genes from the N deficiency transcriptome data (Table S6) for the qRT-PCR expression analysis. The results showed that almost all *GhNPF* members were induced by N deficiency. As shown in Figure 6, *GhNPF4.8, GhNPF4.17, GhNPF5.7*, and *GhNPF8.5* were significantly upregulated, whereas *GhNPF6.12, GhNPF6.14*, and *GhNPF8.5* were inhibited in the roots at low N conditions. Additionally, *GhNPF6.5* and *GhNPF8.3* were upregulated, while *GhNPF1.5* and *GhNPF2.12* were downregulated in shoots under N deficiency. These results were consistent with the changes observed in the RNA-seq data. In shoots, no expression changes were observed for the *GhNPF4.8, GhNPF4.17*, and *GhNPF8.5* genes, suggesting a possible additional development influence.

### 2.7. Silencing of GhNPF6.14 in Cotton

**Figure 7:**
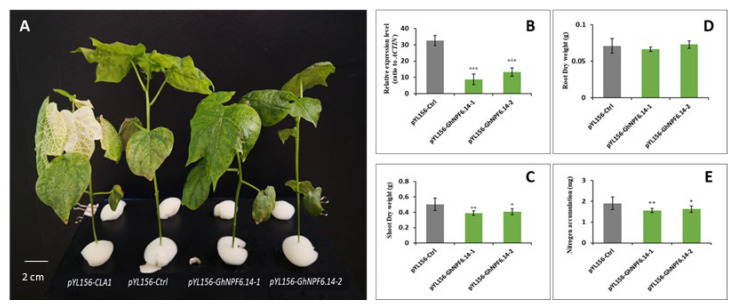
Silencing of *GhNPF6.14* gene by VIGS and plant growth and nitrogen accumulation analysis. (**A**) The phenotype of control and gene-silenced plants with albino appearances. CAL: chlorophyll deficient, *pYL156-Ctrl*: negative control. (**B**) Re-elevate expression level. (**C**) Shoot dry weight. (**D**) Root dry weight. (**E**) Nitrogen accumulation. Error bars indicate the standard deviations of three independent experiments (*/**/*** indicate a significant difference with Ctrl, where * indicates *p* < 0.05, ** indicates *p* < 0.01 and *** indicates *p* < 0.001).

The cotton seedlings were transformed with the *pYL156-CLA1, pYL156-Ctrl*, and *pYL156-GhNPF6.14* vectors. The qRT-PCR method was employed to test the effectiveness of *GhNPF6.14* silencing once the albino phenotype was observed in the true leaves of the treatment plants. The analysis of *PYL156:GhNPF6.14* expression in the shoots showed that the genes were efficiently silenced (Figure 7). Moreover, the root dry weight of *PYL156-GhNPF6.14-*silenced cotton plants have no difference from that of the control plants; however, the shoot dry weight and whole plant N accumulation of *PYL156-GhNPF6.14*-silenced plants were significantly lower than those of the *pYL156-Ctrl* plants. This revealed, to a certain extent, how cotton growth and N absorption and accumulation are impacted by *GhNPF6.14* inactivation.

## 3. Discussion

Nitrogen (N) is one of the most crucial elements for plant growth and development. In terrestrial plants, N uptake, transport, and utilization are facilitated by the NPFs [5,32], especially under N deficiency. Cotton plants are susceptible to N deprivation and, thus, require higher amounts of N fertilizer to compensate for the available N. Various *NPF* genes have been identified in many plant species [18,19,20,21,23,24,25,26,27,28] and are reportedly involved in N uptake, stress responses, and absorption of nutrients. However, the functions of these genes in cotton, particularly under stress conditions such as low N conditions, are still unclear. The current study identified 71, 75, and 150 *NPF* genes in *G. arboreum, G. raimondii*, and *G. hirsutum*, respectively (Table S1). These genes served as candidates for the functional characterization of *NPF* genes in cotton.

Polyploidy has long been a crucial plant diversification mechanism and a key evolutionary driver [33]. The allotetraploid cotton (*G. hirsutum*) originated through a polyploidization event involving the hybridization of the *G. arboreum*-like A-genome ancestor (A2) with the *G. raimondii*-like D-genome ancestor (D5), followed by chromosomal doubling [34]. Previous studies reported the uneven evolution of the At and Dt subgenomes, with structural rearrangements and gene deletions being more frequent in the At subgenome [31]. We found that the *NPF* genes of tetraploid cotton are not the same as the sum of the *NPF* genes in the diploid ones, possibly due to more *GhNPF* genes being lost in the At subgenome than the Dt subgenome.

Duplicated pairings have been shown to frequently form through different mechanisms, such as pseudogenization, neo-functionalization, and sub-functionalization [35]. These suggest testable hypotheses indicating that, whereas sub-functionalized gene copies are subject to purifying selection (Ka/Ks = 1), the neo-functionalized gene copies exhibit positive selection (Ka/Ks > 1) [36]. Most of the duplicated gene pairs in the present study had a Ka/Ks ratio of less than one (Figure 3 and Table S2), indicating that these genes were mostly subject to purifying selection pressure during evolution and preserved their original function. Moreover, the collinear investigation showed that whole-genome duplication event is the mainly power to drive NPF gene family expansion. Conversely, the orthologous gene pairings between the At sub-genome and the A genome had Ka/Ks ratios higher than one, which were higher than those between the Dt sub-genome and the D genome. These suggest that the *NPF* genes of At sub-genomes may have undergone stronger positive selection than Dt sub-genomes during the evolution of tetraploids from diploids.

Eight groups of cotton *NPFs* were identified by phylogenetic analysis (Figure 1 and Figure 2), and their classification was conducted using motif analysis, gene structure, and phylogeny. In addition, the evolutionary analysis showed that the eight cotton subfamilies were similar to those of rice and *Arabidopsis*. Exon–intron and protein structural conservation may, to some extent, represent the functional conservation of the proteins. The analysis of gene structure and conserved motifs revealed that the *GhNPFs* had higher structural consistency. Most *GhNPFs* contained 12 similar motifs, and no unique motifs belonging to a particular subfamily were identified. We also found that some closely related genes exhibited the same motif elimination within the same subfamily. According to gene structure analysis, most *GhNPF* genes have three or four introns, similar to the outcomes reported in *Arabidopsis*, soybean, oilseed, and tea plants. Moreover, *GhNPFs* belonging to the same subfamily had comparable gene architectures and motifs, suggesting that gene structural modifications and conserved motifs may have significantly contributed to the functional diversity of cotton *NPF* genes. The comparable structures and patterns between the *NPF* genes also suggested that there may be biological interactions between genes from the same subfamily. However, the N-terminal ends of proteins exhibited higher variations in motif distribution and type, as demonstrated by the sequence logos of the cytoplasmic protein kinase domains and the extracellular *GhNPFs* domains (Figure S2). The relative diversity of these extracellular domains allows the genes to bind various ligands and sense environmental signals. Moreover, the comparable three-dimensional shapes and subcellular localization of *GhNPF* proteins suggested similarity in their functions.

Cis-elements and trans-acting factors can regulate gene expression by binding the transcription factors of the targeted genes [37]. Thus, cis-acting elements are directly involved in differentiating the targeted genes and regulating their expression. Recent studies revealed that rice development and yield are regulated by the nitrate transporter gene *OsNPF5.16*, whose promoter sequence exhibits natural variation [38]. To understand how *GhNPF* genes are regulated under different environmental conditions, we investigated the cis-acting elements at the promoter region and found that most of them were associated with various hormone and stress responses (Figure S4, Table S3). We identified various *GhNPF* promoters containing cis-elements related to phytohormone responses, including MeJA (85/150 genes), salicylic acid (59/150 genes), and ABA-responsive CRE (96/150 genes). This indicated their possible hormone-producing properties. Plants have been shown to absorb and transport N to adjust to environmental stresses [39]. The tolerance of *Arabidopsis* to drought is regulated by the proteins *AtNPF6.3/AtNRT1.1* [40], while AtNRT1.5 confers salt tolerance to prevent dehydration. The *GhNPF* promoters also contained stress-related cis-elements such as several low temperatures (LTR), drought (MBS), anaerobic conditions (ARE), and wounds (WUN-Motif). These findings indicated that the expression of the *GhNPF* genes might be regulated by many transcription factors.

Abiotic stress negatively impacts plant growth and development, and plants adapt to such conditions by activating various molecular, cellular, and physiological processes [41]. According to the gene expression patterns in response to abiotic stimuli, most *GhNPF* genes are associated with different environmental conditions, including cold, heat, salt, and drought (Figure S6). Moreover, *GhNPF2.12*, *GhNPF2.14*, *GhNPF3.4*, *GhNPF3.8*, *GhNPF4.24*, *GhNPF6.4*, *GhNPF6.8*, *GhNPF6.18*, and *GhNPF6.19* were significantly upregulated under different stress conditions, while *GhNPF5.5* and *GhNPF4.27* were increased under salt stress. Under cold stress, the expression of *GhNPF7.13* was significantly downregulated but increased when exposed to salt, suggesting the significance of *GhNPFs* in plant growth and stress tolerance.

The expression of certain genes in specific tissues can help to understand their roles [42]. In wheat, the extended family members were exposed to nutrition, followed by functional differentiation, to create a genetic foundation for its response to N deprivation in various environments [43]. The primary transport substrate for NPF proteins is nitrates, and more than a third of *Arabidopsis NPF* genes transport nitrates. According to reports, the *Arabidopsis* gene pair *AtNPF4.6/AtNRT1.2* is mostly expressed in the roots to regulate constitutive nitrate uptake [3]. *AtNPF7.3/AtNRT1.5* subsequently transports nitrate from the root to the shoot and mediates nitrate loading in the root xylem [10]. In rice, the lateral roots and stems are the primary sites of *OsNPF7.9* expression, which might increase the N utilization efficiency of the plants [13]. According to RNA-seq analysis, *GhNPF4.8, GhNPF4.17, GhNPF5.7*, and *GhNPF8.5* transcripts were significantly upregulated under low nitrate, whereas *GhNPF6.14, GhNPF6.15*, and *GhNPF6.6* were downregulated under high nitrate conditions in cotton roots. Moreover, the expression levels of *GhNPF1.3, GhNPF5.19, GhNPF8.3*, and *GhNPF8.8* were preferentially increased in the shoots under low N conditions. Some *GhNPF* genes displayed obvious tissue preference (Figure 5, Tables S4 and S6); for example, *GhNPF1.12*, *GhNPF4.8*, *GhNPF4.17*, *GhNPF6.7*, and *GhNPF8.5* were mainly expressed in the stem. Others, such as *GhNPF2.14*, *GhNPF2.15*, *GhNPF3.4*, *GhNPF6.4*, *GhNPF6.8*, *GhNPF6.9*, and *GhNPF7.9,* were expressed in the roots. We also found that the complexity of the expression patterns of *GhNPF* genes and the similarity of those belonging to the same subfamily showed the most of their have similar functionality. Therefore, this study lays the foundation for further functional characterization of *NPF* genes in cotton.

## 4. Materials and Methods

### 4.1. Identification of NPF Genes

The NPF protein family members were identified in the three cotton species (*Gossypium arboreum*, *Gossypium raimondii*, and *Gossypium hirsutum*) based on their homology with the 53 NPF proteins from the *A. thaliana* TAIR database (https://www.arabidopsis.org/). We used the NPF domain (PF00854) as a multiple BLAST query against the cotton database to locate several candidate *NPF* sequences in the three cotton species using HMMER v.3.1 software. Additionally, we examined the putative NPF members using the SMART and CDD databases to determine whether they contained fully or partially conserved motifs. Finally, we obtained 75, 71, and 150 putative *NPF* genes from the *G. raimondii, G. arboreum*, and *G. hirsutum* genomes. The Cottongen (http://www.cottongen.org/) and CottonFGD (https://cottonfgd.org/) databases were used to assemble the CDS, protein, cDNA, and genomic DNA sequences of the *NPFs*. The Ensembl-Plants search tool (http://plants.ensembl.org/) was used to examine the length of the genes and proteins and the number of introns. Furthermore, the MW and PI values of the proteins were determined via the ProtParam tool (https://web.expasy.org/protparam/). The structure of the *NPF* family genes was determined using the TBtools software based on the alignments of their genomic and coding sequences [44].

### 4.2. Chromosomal Location and Sequences and Phylogenetic Analysis

Multiple sequence alignment was performed using DNAMan2.0, and the number of TM domains was obtained via the TMHMM Server v.2.0 (http://www.cbs.dtu.dk/services/TMHMM). The TM domain sequences of the NPFs from *Arabidopsis* and the three cotton species were aligned for the sequence logo analysis, and the obtained alignments were submitted to WEBLOG [45] for logo generation. For phylogenetic analysis, full-length NPF sequences were aligned and used for neighbour-joining (NJ) tree construction using 1000 bootstrap replicates via MEGA 7.2 [46]. Chromosomal location analysis of *NPF* genes was performed using the Map Inspect program.

### 4.3. Analysis of the Conserved Motifs, Gene Structure and Cis-Regulatory Elements

The Multiple Em for Motif Elicitation (MEME) algorithm (http://meme-suite.org/) was used to find the conserved NPF protein motifs, and we ensured that the *p*-value for each motif was less than 1 × 10^−5^. The Newick (NWK) file from the phylogenetic tree analysis was then analyzed using the TBtools program, the Gene Structure Display Server (GSDS) (http://gsds.cbi.pku.edu.cn/), and the MEME MAST program of the MEME suite. Furthermore, the CottonGen database was used to retrieve the 1.5 kb upstream sequences of the initiation codon found in the main *NPF* family genes of *G. hirsutum*, which were then evaluated using the PlantCARE promoter analysis tool.

### 4.4. Evolutionary Analysis of NPF Genes

The homologous *NPF* genes between three *Gossypium* spp. were determined by comparing their coding sequences. The collinearity pairs were retrieved from the *NPF* family using the CIRCOS and TBtools software to create a collinearity map within *NPFs*. Similarly, the TBtools software was used to calculate the synonymous and non-synonymous substitution rates (Ks and Ka, respectively), in which the Ka/Ks ratio > 1, Ka/Ks = 1, and Ka/Ks < 1 indicated positive, neutral, and negative or purifying selection, respectively [47,48]. Each pair of duplicate *NPF* genes was subjected to the calculated selection pressure.

### 4.5. GhNPF-Expression Pattern Analysis

To examine the distribution of *GhNPF* genes in different organs of *G. hirsutum*, we downloaded the raw transcriptome data of the TM−1 cultivar of *G. hirsutum* L. (accession number: PRJNA248163) from the NCBI for analysis [31]. The transcript abundance of *G. hirsutum* NPF genes was calculated as the RPKM (Reads per kilobase of transcript per million mapped reads), and the TBTools program was used to generate the heatmaps and the expression patterns of the *GhNPF* genes presented in Tables S5 and S6.

### 4.6. N deficiency Treatments and the qRT-PCR Analysis

The N deficiency experiment was set up in the greenhouse at CRI, CAAS, and Anyang, China. After germination, *G. hirsutum* L. acc. TM−1 (Upland Cotton Genetic Standard Line) seedlings were transplanted in a plastic container (7 L) in a growth condition of 16/8 h light/dark cycle, 28 °C temperature, and 60% relative humidity as mentioned in our previous study [49,50]. For N deficiency treatments, the third-leaf stage seedlings were transferred to a 0.2 mM nitrate solution, and the 2.0 mM nitrate solution was used as the control. Moreover, in the low N treatment, a total concentration of 1.8 mM CaCl_2_ was added to equalize calcium concentration. The solutions were replaced on a weekly basis and aerated with an electric pump. After 28 d, four plants from each treatment were harvested for RNA-seq analysis with three biological replications. We selected 16 representative *GhNPF* family genes from the N deficiency transcriptome data for designing qPCR-specific primers using the Real-time PCR (TaqMan) Primer and Probes Design Tool (Real-time PCR Primer Design—Real-time PCR Probe Design—GenScript). The primer sequences are shown in Table S7. Total RNA was extracted using the EASYspin plus Plant RNA Kit and reverse transcribed using TransStart^®®^ Top Green qPCR SuperMix (+DyeII) for cDNA synthesis [51]. The housekeeping gene, β-Actin, served as an internal control for the qPCR analysis, and the relative expression of the genes was determined using the 2^−ΔΔCt^ method [52,53].

### 4.7. VIGS Treatment and Measurement of N Concentration

To silence *GhNPF6.14* through the VIGS (virus-induced gene silencing) system. A 300-bp purified gene segment was inserted between the EcoRl and XbaI sites of the pYL156 vector using the primer sequences GTGAGTAAGGTTACCGAATTCATGTCCCTCCCTGTAACACAAGG-F, TCCCCATGGAGGCCTTCTAGACTAATTCAATCCCTTCATCCGC-R. We transformed the recombinant plasmid into *Agrobacterium* LBA4404 competent cells to create recombinant *Agrobacteria* carrying *pYL156-GhNPF6.14*. Silencing the chlorophyll biosynthesis gene *pYL156-CLA1* affected the synthesis of chlorophyll in cotton leaves. The experiment was considered successful when the albino phenotype was observed on the *pYL156-CLA1* cotton plant. The infiltrated plants were maintained at 25 °C for effective viral infection and spread. The plant growing conditions same as the previous experiments, and the 2.0 mM nitrate solution was used. Three plants from each treatment were harvested for the next experiment’s analysis with three biological replications. The Kjeldahl method was used to measure the N concentrations in plant tissues. Briefly, the shoot and root samples were dried and crushed into a fine powder. Approximately 0.2 g of each sample was weighed and digested with H_2_SO_4_-H_2_O_2,_ and the amount of N in each sample was determined using the AutoAnalyzer III (AA3).

## 5. Conclusions

This study identified the members of the *NPF* gene family in three cotton species. We performed evolutionary connections, structural, subcellular localization, cis-elements, and expression pattern analyses of the identified *NPF* genes to determine the potential roles of *NPF* genes in cotton under various abiotic stress conditions. The collinearity research revealed that tandem and segmental duplications contributed to the *NPF* gene family variation in cotton. According to the expression analysis, *GhNPF* genes may be crucial for cotton growth and development in stressful conditions, particularly low-N environments. These results provide the basis for future research on the molecular mechanisms underpinning the *NPF* gene activities in N utilization to enhance nitrogen use efficiency (NUE) and cotton productivity.

## Data Availability

Data are contained within the article or supplementary materials.

## Supplementary Materials

The following supporting information can be downloaded at: https://www.mdpi.com/article/10.3390/ijms232214262/s1.
