# Supplementary material for: Identification and Expression Analysis of the NPF Genes in Cotton
Source: Int J Mol Sci. 2022 Nov 17;23(22):14262. doi: 10.3390/ijms232214262 (PMC9692789; doi:10.3390/ijms232214262)
Supplement: Supplementary file 1 [file ijms-23-14262-s001.zip › Supplement Fig S1-S6.pdf]

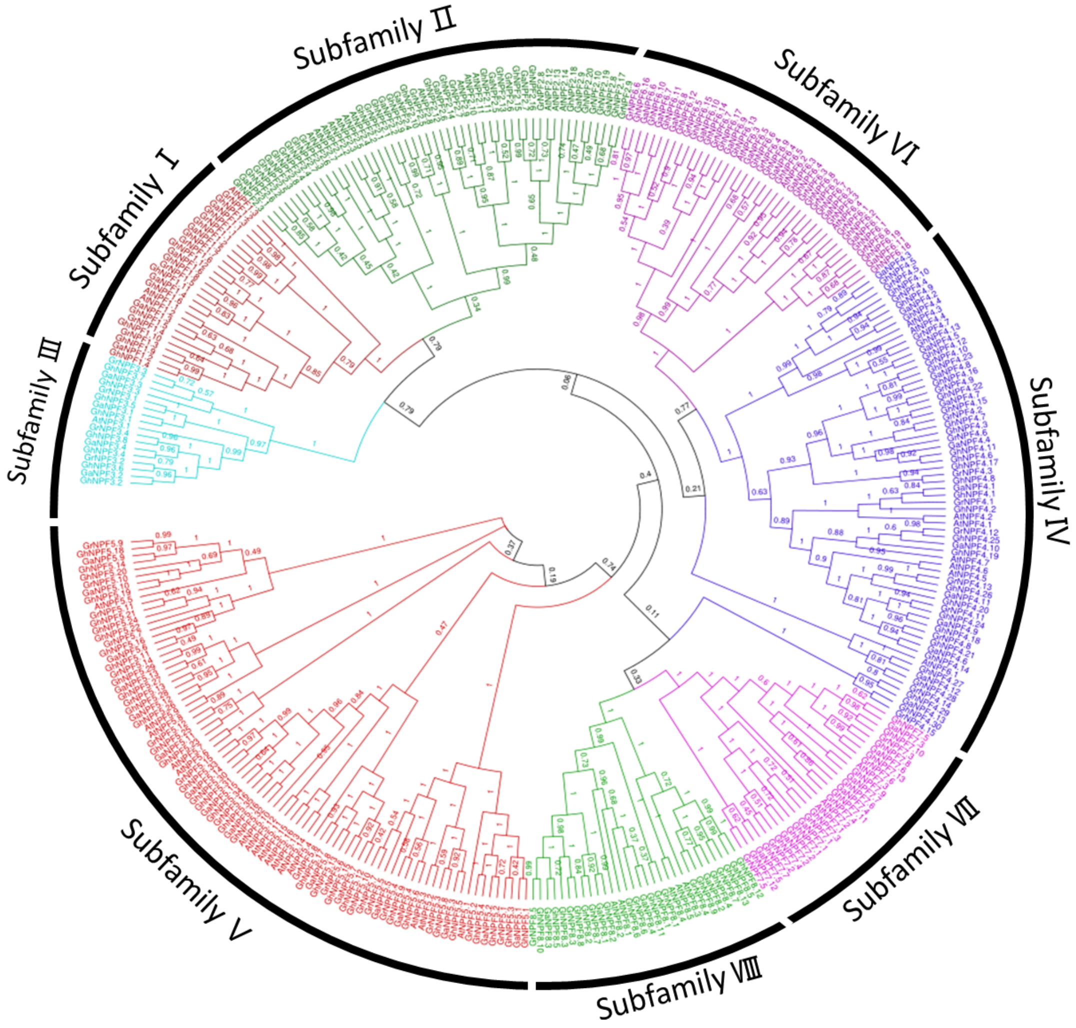

Figure S1. Phylogenetic tree and subgroup classification of NPF proteins in *A. thaliana*, *Gossypium hirsutum*, *G. raimondii* and *G. arboreum*. The numbers at nodes of the phylogenetic tree indicate the bootstrap values expressing branching probability per 1000 replicates; the bootstrap values of the confidence levels are shown as percentages.

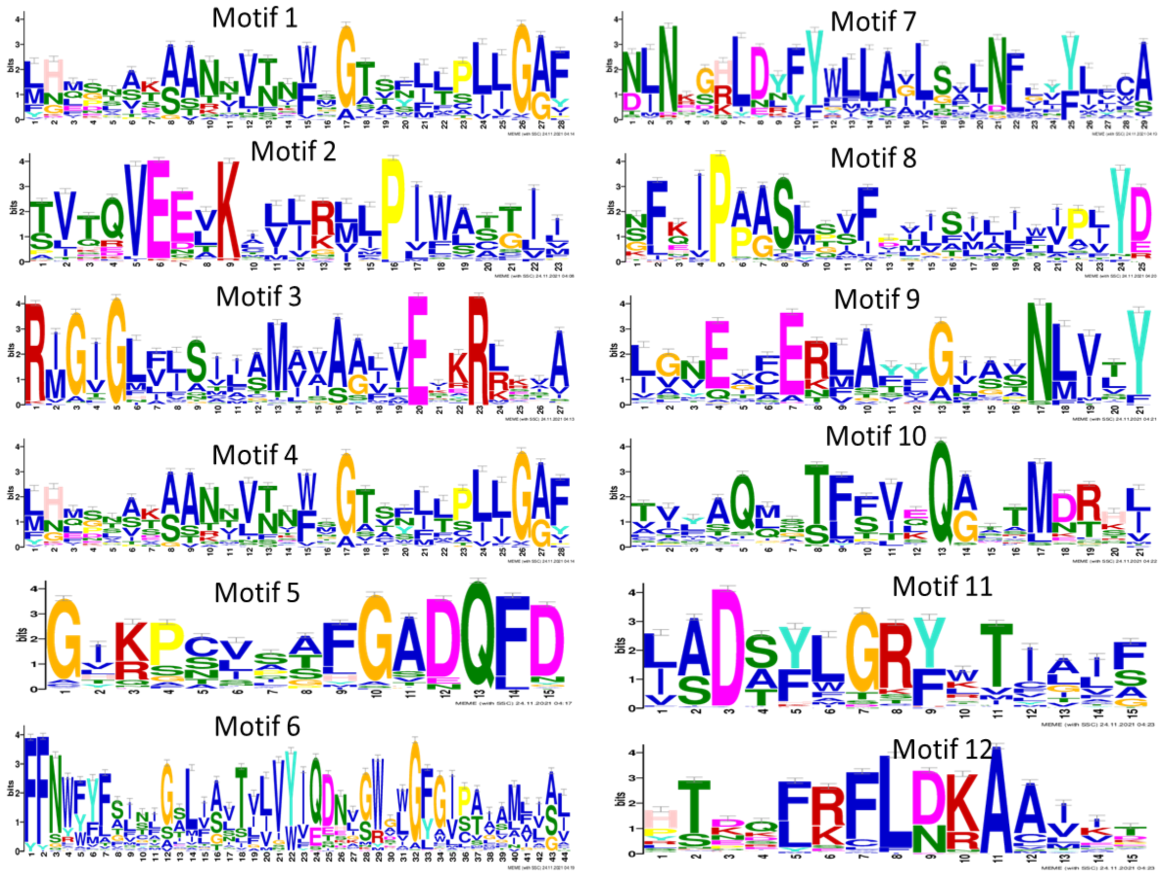

Figure S2. Sequence logo of the *GhNPFs* conserved-domain.

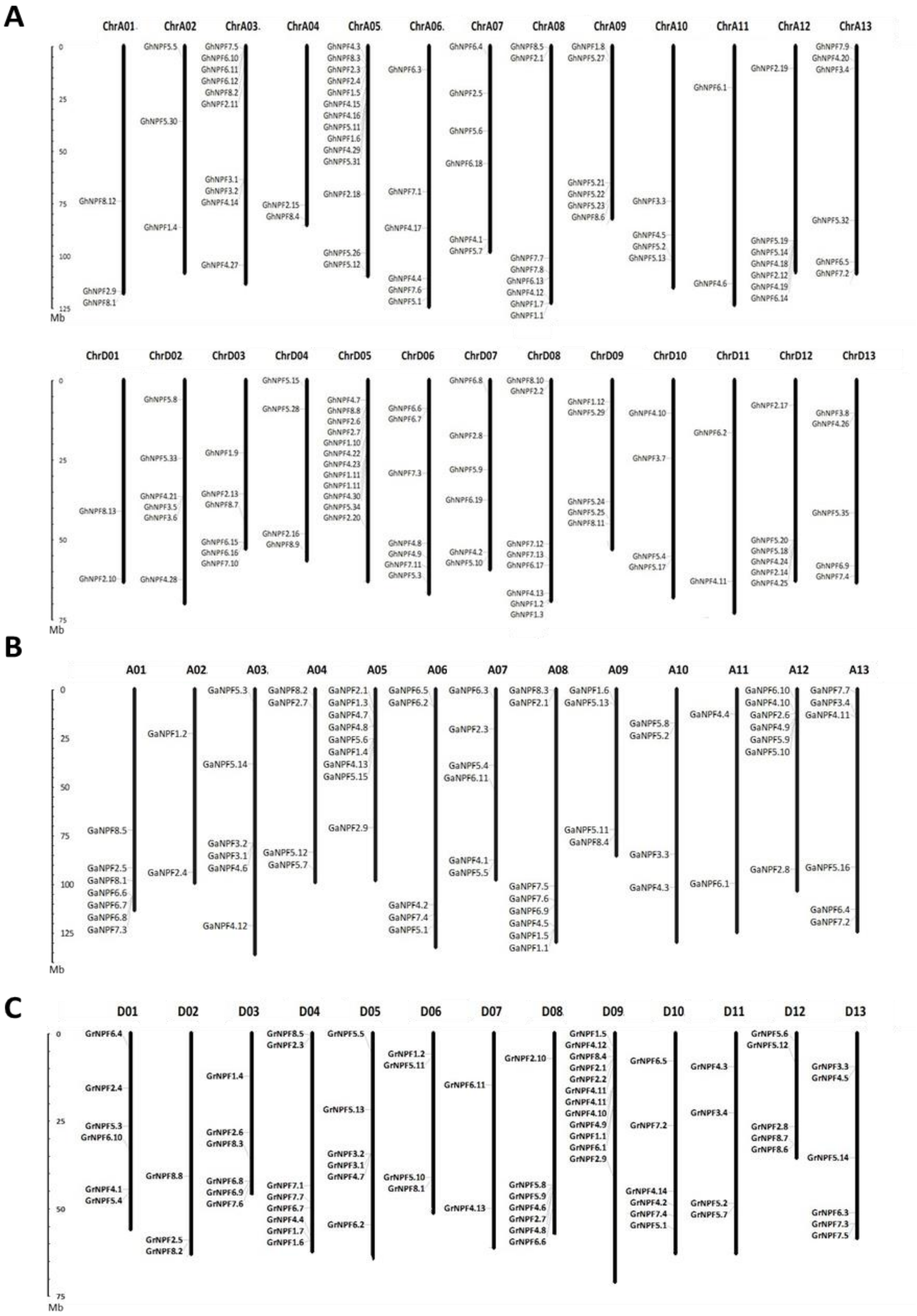

Figure S3. Chromosome distribution of *NPF* genes in *G. hirsutum* (A), *G. raimondii* (B) and *G. arboreum* (C). Scale on left is in Mb. Chromosome numbers are indicated on top of bar.

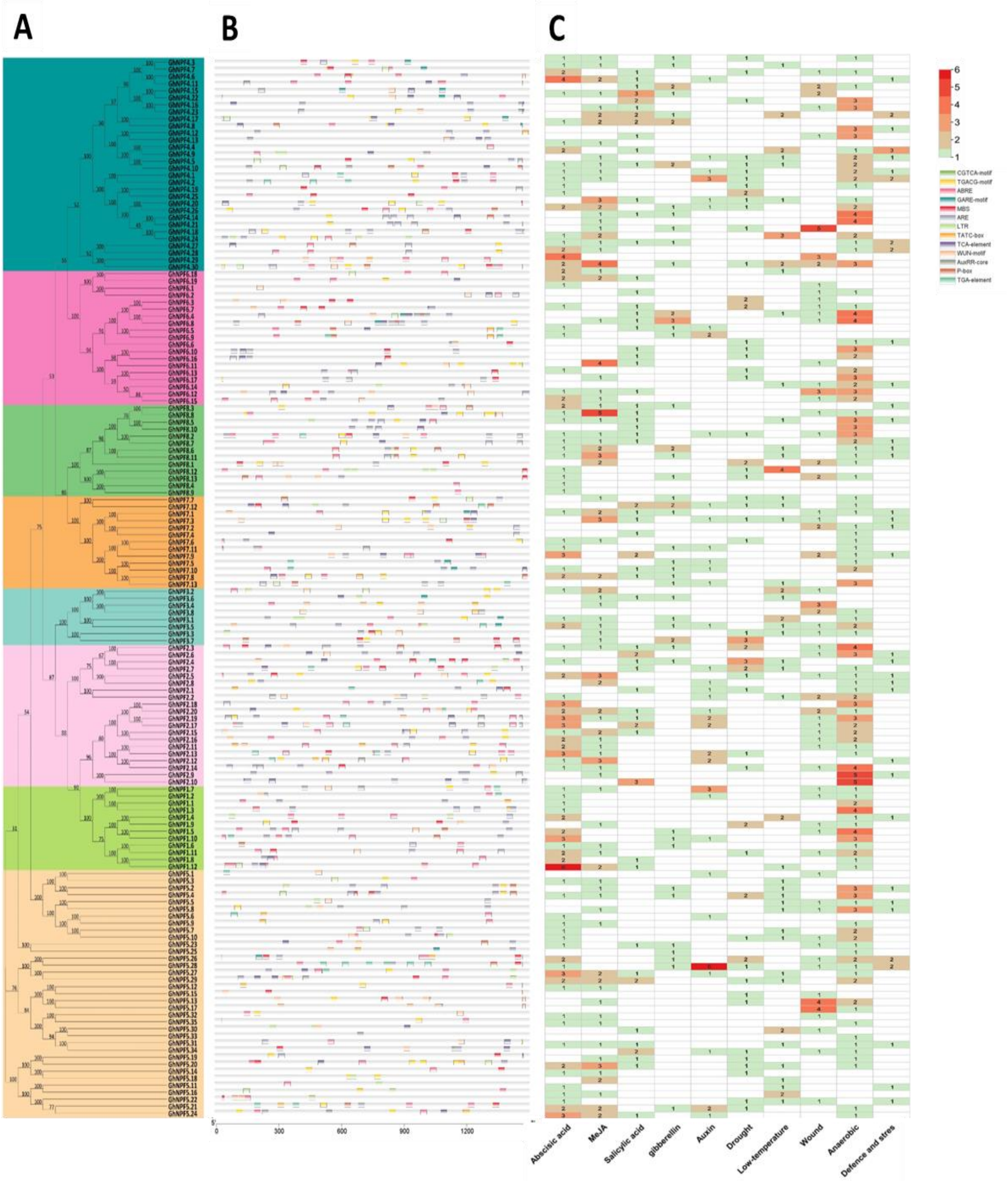

Figure S4. Analysis of cis-elements in *GhNPF* promoters. (A) Phylogenetic tree of *GhNPFs*. (B) Cis-elements in promoters of *GhNPF* gene family members. (C) Frequency of cis-elements in the promoter region of *GhNPF* genes. The number in colored box represents the number of cis-acting elements.

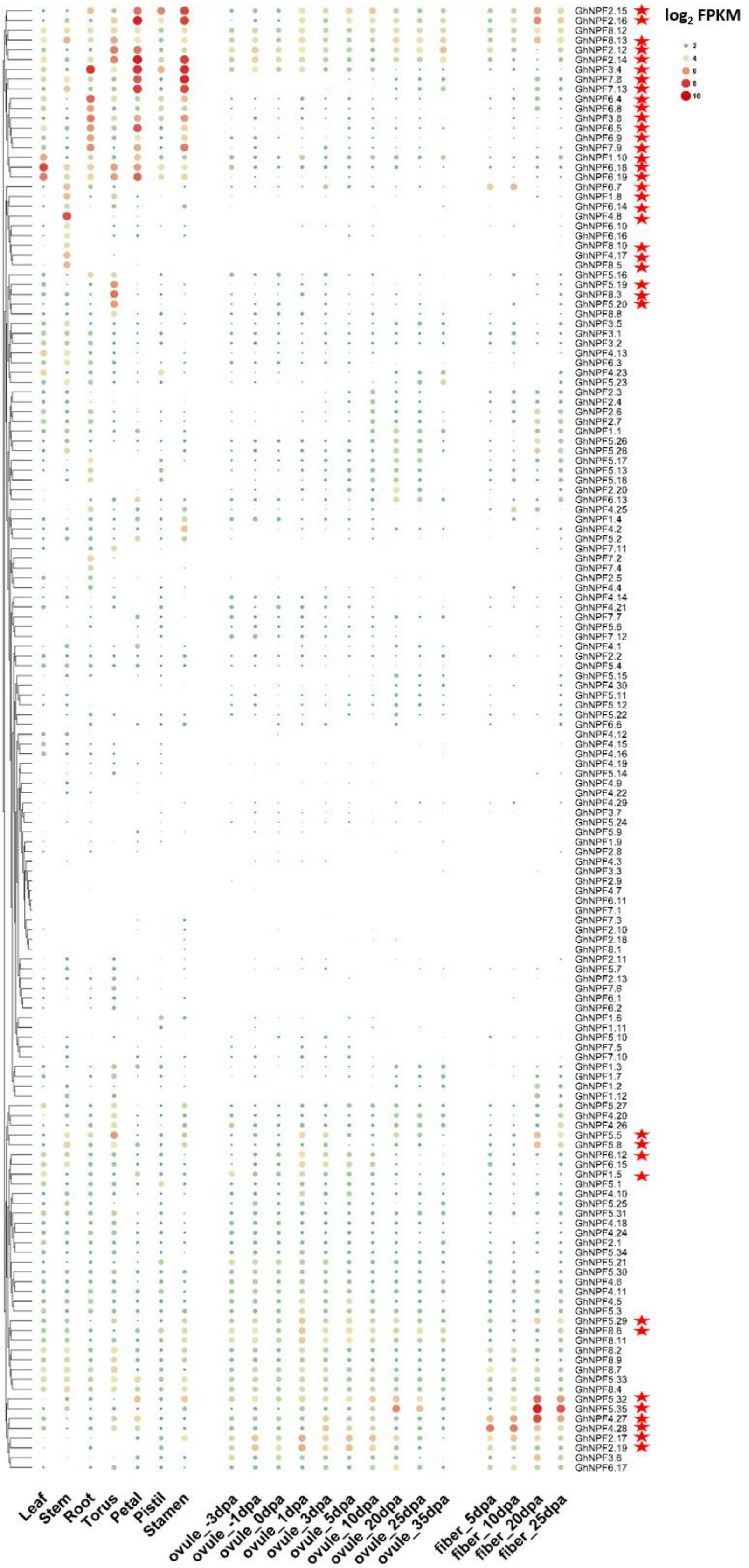

Figure S5. Expression patterns of *GhNPF* family genes in different tissues. The expression data were obtained from RNA-seq data and shown as log_2 FPKM. *Asterisks* marked *GhNPF* genes selected for N deficiency treatment candidate genes.

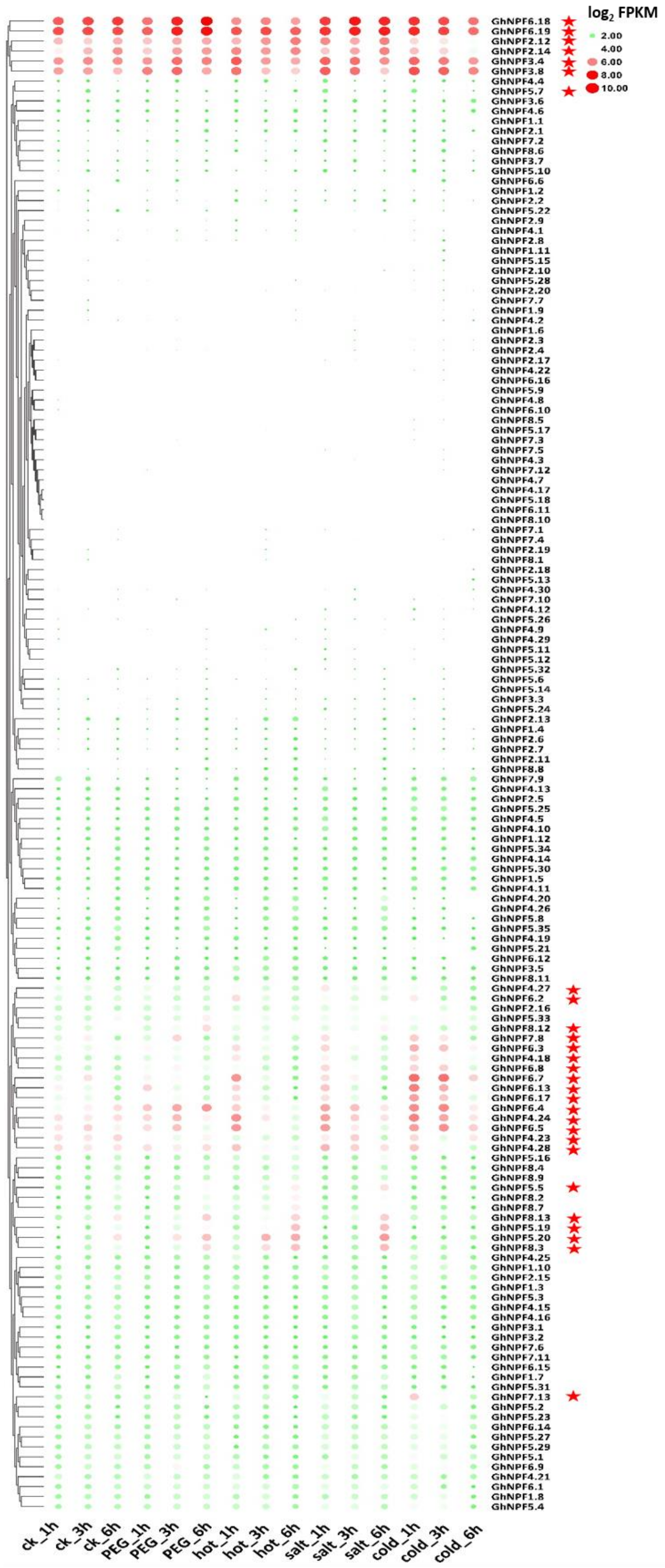

Figure S6. Differentially expression level of *GhNPF* gene family members under cold, hot, salt and PEG stress. The expression data were obtained from RNA-seq data and shown as log_2 FPKM. *Asterisks* marked *GhNPF* genes selected for N deficiency treatment candidate genes.
