# Supplementary material for: Identification and Expression Analysis of the NPF Genes in Cotton
Source: Int J Mol Sci. 2022 Nov 17;23(22):14262. doi: 10.3390/ijms232214262 (PMC9692789; doi:10.3390/ijms232214262)
Supplement: Supplementary file 1 [file ijms-23-14262-s001.zip › Supplement Table S7.pdf]

Table S7. Primer sequences used in qRT-PCR.

Gene	Forward primer (5'-3')	Reverse primer (5'-3')
GhNPF1.5	CAATTTCACCCCTATATTG	TCTGTGTCATTAGGTCGCAG
GhNPF2.12	GATTCTTGGACAAAGCAGCT	ACAATACATGATTTGTGATGC
GhNPF3.4	GTATCAATACTTGTGGCTGTC	ACACACTGACCTGTACCAAAC
GhNPF4.8	CAGATAGTGAATCGTGCAACAAA	GACCTGTACTTATAACGCGAAG
GhNPF4.17	CACTCACCTTCAACGTATAG	AGAGCCAGAAGACACTGATAG
GhNPF4.27	TTGCGGGTTTGATAGGGATAA	GACGCAGGGTCTTATACCTG
GhNPF5.7	CAGCTTGTCTCACAGCGTT	TCTTTGAAGCAAGGTGATGC
GhNPF5.35	ACTACATCCAGGAAAACCT	TCTTCATCCCCTTTGACACT
GhNPF6.5	GTCCAGTTACAAGGGTAGAG	GAAATGTCCTATGGTTCTGT
GhNPF6.12	AACAGTTCCGTTTCTTGGAT	TACAGTGGTTGCCCATATGG
GhNPF6.14	CTGTGCTTGTGCAATCGTGA	AATGCCTCTTCTTCCACGCAA
GhNPF6.18	AACTGTCGTATGGAGGGTTC	GTGTAGGGAACCTTTCGGTTA
GhNPF7.8	TAGTTGGTGCTTTCCTCAGTG	AAGTATGATGATAGCGACAG
GhNPF8.3	TCAATGAGGAGTTTGTGGTC	GGTTGTCCGGTATCCAGCCA
GhNPF8.5	AGGTAATGAGGGTTGTGAAAG	TGTAATGTAACATGTTCCACT
GhNPF8.13	GTTCCCATCTATGATAGGAT	CTGATCTCCACTACAGCTGC
